# Abnormal glycosylation in Joubert syndrome type 10

**DOI:** 10.1186/s13630-017-0048-6

**Published:** 2017-03-23

**Authors:** Megan S. Kane, Mariska Davids, Michelle R. Bond, Christopher J. Adams, Megan E. Grout, Ian G. Phelps, Diana R. O’Day, Jennifer C. Dempsey, Xeuli Li, Gretchen Golas, Gilbert Vezina, Meral Gunay-Aygun, John A. Hanover, Dan Doherty, Miao He, May Christine V. Malicdan, William A. Gahl, Cornelius F. Boerkoel

**Affiliations:** 1 NIH Undiagnosed Disease Program, Common Fund, Office of the Director, and National Human Genome Research Institute, National Institutes of Health, Bethesda, MD USA; 2 Inova Translational Medicine Institute, Inova Health System, Falls Church, VA USA; 3 National Institute of Diabetes and Digestive and Kidney Diseases, National Institutes of Health, Bethesda, MD USA; 4 Department of Pediatrics, University of Washington, Seattle, WA USA; 5 The Michael J Palmieri Metabolic Laboratory, Children’s Hospital of Philadelphia, Philadelphia, PA USA; 6 Children’s National Medical Center, Washington, DC USA; 7 Medical Genetics Branch, National Human Genome Research Institute, National Institutes of Health, Bethesda, MD USA; 8 Office of the Clinical Director, National Human Genome Research Institute, National Institutes of Health, Bethesda, MD USA; 9 Johns Hopkins University School of Medicine, Department of Pediatrics and McKusick-Nathans Institute of Genetic Medicine, Baltimore, MD USA; 10 Department of Medical Genetics, University of British Columbia, Vancouver, BC Canada

**Keywords:** Joubert syndrome, Ciliopathy, Glycosylation, Molar tooth sign, CMP-sialic acid

## Abstract

**Background:**

The discovery of disease pathogenesis requires systematic agnostic screening of multiple homeostatic processes that may become deregulated. We illustrate this principle in the evaluation and diagnosis of a 5-year-old boy with Joubert syndrome type 10 (JBTS10). He carried the OFD1 mutation p.Gln886Lysfs*2 (NM_003611.2: c.2656del) and manifested features of Joubert syndrome.

**Methods:**

We integrated exome sequencing, MALDI-TOF mass spectrometry analyses of plasma and cultured dermal fibroblasts glycomes, and full clinical evaluation of the proband. Analyses of cilia formation and lectin staining were performed by immunofluorescence. Measurement of cellular nucleotide sugar levels was performed with high-performance anion-exchange chromatography with pulsed amperometric detection. Statistical analyses utilized the Student’s and Fisher’s exact *t* tests.

**Results:**

Glycome analyses of plasma and cultured dermal fibroblasts identified abnormal *N*- and *O*-linked glycosylation profiles. These findings replicated in two unrelated males with *OFD1* mutations. Cultured fibroblasts from affected individuals had a defect in ciliogenesis. The proband’s fibroblasts also had an abnormally elevated nuclear sialylation signature and increased total cellular levels of CMP-sialic acid. Ciliogenesis and each glycosylation anomaly were rescued by expression of wild-type *OFD1*.

**Conclusions:**

The rescue of ciliogenesis and glycosylation upon reintroduction of WT *OFD1* suggests that both contribute to the pathogenesis of JBTS10.

**Electronic supplementary material:**

The online version of this article (doi:10.1186/s13630-017-0048-6) contains supplementary material, which is available to authorized users.

## Background

Joubert syndrome (JBTS) is a rare genetic condition characterized by hypotonia, developmental delay, cerebellar dysfunction, a neuroradiologic “molar tooth sign” (MTS), and variable involvement of other systems [1, 2]. JBTS is a ciliopathy, and mutations in more than 30 genes encoding products localizing primarily to and around the cilia are implicated in its pathogenesis [3, 4]. The genetic locus associated with JBTS type 10 (OMIM 300804) is *OFD1*; mutations in this gene also cause the X-linked dominant disorder orofaciodigital syndrome I (OFDI, OMIM 311200), the X-linked recessive disorder Simpson–Golabi–Behmel syndrome, type 2 (SGBS2, OMIM 300209), and an X-linked form of retinitis pigmentosa (RP23, OMIM 300424) [5].

The ciliary function of the OFD1 protein has not been fully elucidated, although it does play a role in primary ciliogenesis and regulation of ciliary length [6, 7]. Loss of *OFD1* in animals causes defects in left/right axis development, ciliogenesis, and neurological development [8–10]. These phenotypes are consistent with features observed in other ciliopathy disorders and reflect the role of primary cilia in the Shh, Wnt, and planar cell polarity (PCP) signaling pathways [4]. In the centrosome, OFD1 interacts with lebercilin, a key protein in the pathogenesis of Leber congenital amaurosis [11, 12]. Based upon its nuclear localization, there is a hypothesis that OFD1 plays a role in chromatin remodeling [13].

The National Institutes of Health Undiagnosed Disease Program (NIH UDP) evaluates individuals with rare and undiagnosed diseases and an indication of a genetic etiology. Physicians across multiple specialties consult on each patient to obtain a full phenotypic description. In addition to targeted diagnostic tests, the NIH UDP implements broader, agnostic screens of exome, glycome, and metabolome.

Herein we report the evaluation of a 5-year-old boy with Joubert syndrome secondary to mutation in *OFD1.* His cultured dermal fibroblasts had impaired ciliogenesis, and his plasma and cultured dermal fibroblasts had abnormal *N*- and *O*-linked glycosylation profiles. Glycomic evaluation of two unrelated individuals with JBTS10 detected similar plasma and fibroblast *N*- and *O*-linked glycosylation profiles. Cultured fibroblasts from the proband also had increased CMP-sialic acid. Overexpression of wild-type *OFD1* cDNA rescued both the glycosylation and ciliary phenotypes. We posit, therefore, that glycosylation anomalies are a previously undocumented contributor to the pathogenesis of JBTS10.

## Methods

### Patient enrollment and consent

The proband, UDP-3331, was evaluated in the National Institutes of Health Undiagnosed Diseases Program (NIH UDP) and was enrolled in clinical protocol 76-HG-0238, approved by the NHGRI Institutional Review Board. His mother, UDP-4786, provided informed consent. NIH-452 was enrolled in protocol “Clinical and Molecular Investigations into Ciliopathies” (Trial NCT00068224); his legal guardian provided informed consent. Both protocols conform to the 1964 Helsinki Declaration and subsequent amendments standard for patient protection.

### Variant identification and Sanger sequencing

Genomic DNA was extracted from peripheral whole blood of UDP-3331 and UDP-4786 using the Gentra Puregene Blood Kit (Qiagen, Valencia, CA) and an AutoGen FlexStar with standard procedures. The DNA of all family members was subjected to an integrated set of genomic analyses including high-density single-nucleotide polymorphism (SNP) arrays and whole exome sequencing (WES) as described previously [14–17]. WES was performed on these individuals using the Illumina HiSeq2000 platform and the TrueSeqV2 capture kit (Illumina, San Diego, CA). Sample library preparation, sequencing, and analysis were performed using the standard NIH Intramural Sequencing Center (NISC) pipeline [18]. Sequence data were aligned to human reference genome (hg19) using Novoalign (Novocraft Technologies, Selangor, Malaysia). To test for copy number variants and to form segregation BED files for exome analysis, Omni Express 12 (hg19) SNP arrays were run on genomic DNA from all family members as described [14]. Variants listed in the Variant Call Files (VCFs) were filtered based on rarity, Mendelian segregation, and predicted deleteriousness on the Cartagenia platform (Cartagenia N.V., Leuven, Belgium).

Exome variants were filtered using ExAC release 0.3 population frequency data (MAF < 0.02, 95% confidence interval; homozygote count ≤25) and UDP founders cohort population frequency data (variant allele count <8). Variants were prioritized based on coding effect (nonsynonymous, frameshift, stopgain, stoploss, startloss, inframe), proximity to splice sites (within 20 base pairs of a canonical splice site into the intron, or 5 base pairs into the exon), and CADD v1.3 Phred scores. Using the Integrative Genome Viewer (https://www.broadinstitute.org/igv/home), we assessed the quality of alignment and genotype call of variants. The single-nucleotide deletion identified by exome sequencing was confirmed using Sanger sequencing of the genomic DNA from both UDP-3331 and UDP-4786; the forward primer used was 5′-AGTAGGGTACAGTTCAAGGAGTGG-3′ and the reverse primer was 5′-ATTCTGTGCAGCCTTTCCCAC-3′. DNA amplification was performed with Platinum *Pfx* DNA polymerase (ThermoFisher Scientific, Waltham, Massachussets) and standard thermal cycling protocol with an annealing temperature of 55 °C for a total of 30 cycles.

### *OFD1* cloning

Wild-type (WT) cDNA expression constructs were made by PCR amplification of the *OFD1* cDNA sequence from a sequence-verified TrueORF Gold clone (RC220154, Origene, Rockville, Maryland). The PCR product was cloned into pENTR/D-TOPO vector (ThermoFisher Scientific) and subcloned into pLENTI6.3/V5-DEST (ThermoFisher Scientific) using the Gateway LR Clonase II enzyme (ThermoFisher Scientific). Clones were verified by Sanger sequencing.

### Lentiviral infection and rescue

Lentivirus was generated by transfecting 293FT cells with the pLENTI6.3-OFD1 construct and the ViraPower HiPerform Lentiviral Gateway expression kit (ThermoFisher Scientific) as per the manufacturer’s protocol. Lentiviral culture supernatant was applied to patient and adult WT control cells in the presence of polybrene (10 µg/mL, EMD Millipore, Billerica, Massachusetts). Stable integration of the *OFD1* construct was selected by addition of 5 µg/mL blasticidin (ThermoFisher Scientific) to the medium and confirmed by qPCR and immunoblotting. Subsequently, retention of the integrated active *OFD1* construct was selected by growth in medium supplemented with 2.5 µg/mL blasticidin.

### Primary fibroblast isolation and culture

Primary dermal fibroblasts from UDP-3331 were derived from a forearm skin punch biopsy. Additional fibroblast lines were obtained from ATCC (ATCC-PCS-201-012, Manassas, Virginia) and Coriell Repository [GM00409 (7 y.o. male) and GM08398 (8 y.o. male), Camden, New Jersey]. The established fibroblast lines were cultured in growth medium, consisting of Dulbecco’s Modified Eagle Medium (DMEM) with 1 g/L glucose (low glucose DMEM), 10% heat inactivated fetal bovine serum (HI FBS, ThermoFisher Scientific), and 1% antibiotic/antimycotic (ThermoFisher Scientific). Culture of primary fibroblasts for glycome analysis utilized α-MEM (Cellgro, Mediatech, Manassas, Virginia) supplemented with 15% HI FBS and 1% antibiotic/antimycotic for 24–48 h prior to cell harvest.

### RNA isolation and qRT-PCR analysis

RNA extraction from cultured dermal fibroblasts was performed as per the manufacturer’s protocol using the Qiagen RNeasy Mini kit (Qiagen, Hilden, Germany) with on-column DNA digestion. Equal amounts of RNA were reverse-transcribed from each cell line using the OmniScript cDNA first-strand synthesis kit (Qiagen). SYBR Green reagent (Qiagen) was used to amplify *OFD1* and *GAPDH* for a ∆∆Ct relative quantification of *OFD1* expression on an ABI7500 Fast RealTime PCR machine with the Melt Curve option. Quantification and quality control parameters were according to the standard manufacturer’s settings. Primers used for *OFD1* were 5′-CGGAGCAGAAAGTGGGTCTTT-3′ and 5′-TGGCATGTTCCCTGCAGATT-3′; primers for *GAPDH* were 5′-TGCACCACCAACTGCTTAGC-3′ and 5′-GGCATGGACTGTGGTCATGAG-3′.

### Immunoblotting

Fibroblasts were cultured to near 100% confluence, rinsed twice with PBS, and harvested by in-dish lysis with RIPA buffer (Sigma-Aldrich, St. Louis, Missouri) supplemented with 1× complete Ultra Protease Inhibitor Cocktail (Sigma-Aldrich). Total protein concentration of the lysates was determined using the DC Protein Assay (BioRad, Hercules, California) and equal amounts of protein were run for each sample on SDS-PAGE gels (4–12% gradient, BioRad) in SDS–Tris–glycine buffer. Proteins were transferred using a standard wet-transfer method to Immobilon-FL PVDF membranes in Tris–glycine buffer with 20% methanol. Blocking was performed with Odyssey blocking buffer in PBS (LiCor, Lincoln, Nebraska); primary antibodies were incubated overnight at 4 °C in fresh blocking solution. Membranes were washed three times with PBS-Tween 20 (0.1%) and incubated with Odyssey near-IR conjugated secondary antibodies for 1 h in Odyssey blocking buffer with Tween-20 (0.1%) and SDS (0.2%). Prior to imaging on a LiCor Odyssey CLx, membranes were washed three times with PBS-Tween 20 and rinsed once with diH_2_O. Band quantification and analysis were performed using the CLx imaging software (LiCor). Antibodies used were anti-OFD1 (1:1000, TA308968, Origene), anti-α-tubulin (1:5000, ab7291, Abcam; Cambridge, Massachusetts), anti-rabbit IR800 (1:10,000, 925–32,211, LiCor), and anti-mouse IR680 (1:10,000, 925–68,020, LiCor).

### Immunofluorescence of cultured fibroblasts

Staining of cultured fibroblasts, either with antibodies or lectins, utilized a similar protocol with minor variations detailed below. Cultured cells were fixed with 4% paraformaldehyde in phosphate-buffered saline (PBS) at room temperature for 10 min, or in ice-cold 100% methanol at −20 °C for 15 min, or a combination of both. The fixed cells were washed three times with PBS. Paraformaldehyde-fixed cells were permeabilized with 0.5% NP-40 detergent in PBS at room temperature for 10 min then washed three times in PBS. The fixed and permeabilized cells were then incubated with a blocking buffer for 1 h. Primary staining reagent(s) were diluted in blocking buffer and applied for 1–2 h at room temperature or overnight at 4 °C. The cells were then washed with PBS three times. The cells were then incubated with secondary detection reagents with fluorophore conjugates diluted in blocking buffer for 1 h at room temperature. After washing the cells three times with PBS, the coverslips were mounted onto slides with the indicated mounting media prior to imaging.

### OFD1 localization and cell morphology analysis

Cells were grown on 12-mm round coverslips to 50–70% confluence at the time of fixation. Cells were fixed either in paraformaldehyde or methanol, depending upon manufacturers’ recommendations for the primary antibodies. The blocking reagent used was 4% BSA (bovine serum albumin) in PBS, and the primary antibodies used were OFD1 (1:100, Origene), ɣ-Tubulin (1:1000; T5326, Sigma-Aldrich), α-tubulin (1:2000, ab7291, Abcam), PCM1 (1:100, sc-50164, SantaCruz Biotechnology, Dallas, Texas), GM-130 (1:250, 610822, BD Biosciences, San Jose, California), TGN-46 (1:200, GTX74290, Genetex, Irvine, California), or KDEL (1:100, ab12223, Abcam). Alexa Fluor conjugated secondary antibodies used were Alexa488 Donkey anti-Rabbit, Alexa555 Donkey anti-Mouse, and Alexa647 Donkey anti-Rabbit (ThermoFisher Scientific). Where indicated, DNA was counterstained with Hoechst 33342 (ThermoFisher Scientific) and coverslips were rinsed with PBS. The mounting medium was ProLong Gold antifade mounting medium (ThermoFisher Scientific) and the slides were imaged using a 20× or 40× objective on a Zeiss LSM 700 confocal laser-scanning microscope (Carl Zeiss Microscopy, GmbH, Jena, Germany).

### Cilia measurements

Fibroblasts were grown to 80% confluence and then serum starved for 48 h. Cells were fixed with 4% paraformaldehyde for 5 min at room temperature followed by ice-cold methanol for 4 min at −20 °C. The blocking buffer used was PBS containing 10% normal donkey serum, 1% BSA, and 0.1% triton X-100 for 1 h. Fixed cells were incubated overnight with primary antibodies (1:1000–1:2000, mouse anti-acetylated Tubulin, T6793, SigmaAldrich; 1:200, goat anti-γ tubulin, sc-7396, Santa Cruz). Secondary antibodies used were AlexaFluor conjugated secondary antibodies (Life Technologies). Coverslips were mounted using Fluoromount G with 4′,6-diamidino-2-phenylindole (DAPI) (Southern Biotech, 0100-20, Birmingham, Alabama).

Fourteen image z-stacks with 0.3 mm spacing were taken with a CoolSNAP HQ2 digital monochrome camera (Photometrics, Tucson, Arizona, USA) through a Marianas live cell imaging system (Intelligent Imaging Innovations, Denver, Colorado, USA) using a Plan Apochromat 63X, 1.4 NA oil objective.

Percentage of ciliated cells and length measurements were made in FIJI on 16-bit sum projection images of z-stacks. Cilia were defined as linear acetylated tubulin-positive structures >1-μm long with a punctate gamma-tubulin signal at one end. The percentage of ciliated cells was determined by dividing the number of ciliated cells by the number of DAPI-positive nuclei in the same images. To measure cilium length, we manually painted a mask over each cilium (defined by acetylated tubulin) with a 3 pixel-wide brush, skeletonized the cilium mask, and applied the maximum branch length function.

### Lectin staining of cultured fibroblasts

Biotinylated Maackia amurensis lectin II (MAL II), fluorescein Sambucus nigra (elderberry) bark lectin (SNA), and rhodamine Peanut Agglutinin (PNA) were obtained from Vector Laboratories (Burlingame, California). Cells were seeded at 0.5 × 10^5^ per 12-mm round coverslip and cultured overnight. The cells were fixed with paraformaldehyde or methanol as indicated in the text. Blocking was performed with 4% BSA in PBS, followed by primary incubation with MAL II, SNA, and/or PNA lectins (20 µg/mL). Detection of biotinylated MAL II required secondary incubation with Streptavidin AlexaFluor 350 (S-11,249, ThermoFisher Scientific). In cells without MAL II staining, DNA was stained with Hoechst 33342 (ThermoFisher Scientific). Coverslips were mounted on slides using ProLong Gold anti-fade reagent then imaged using a 20× objective on a Zeiss LSM 700 confocal laser-scanning microscope. Image analysis was performed using ImageJ software [19].

### Neuraminidase treatment and cilia measurement

Adult WT fibroblasts were plated directly on coverslips at 0.5 × 10^5^ cells per 12-mm coverslip density, allowed to adhere overnight, and then grown for additional 48 h in the absence of serum. Half of the coverslips were washed with Hank’s Balanced Salt Solution (HBSS) and then incubated for 1 h at 37 °C in 1 mU/mL neuraminidase (Sigma, N2133) dissolved in HBSS. Untreated cells were left in serum-free media. Cells were subsequently washed three times in PBS and then fixed using ice-cold methanol. Staining for cilia utilized anti-ARL13B (1:250, ProteinTech, Rosemont, Illinois) and anti-gamma tubulin (1:250, Sigma-Alridch) with Hoechst 33342 DNA counterstaining. DNA counterstaining in conjunction with lectin staining, which was performed as stated above, utilized the far-red dye DRAQ5 (Abcam). Cilia were defined as above and measured with FIJI software; the percentage of ciliated cells was defined as above.

### Glycome analysis

Primary dermal fibroblasts were cultured and processed to release *N*- and *O*-linked glycans as previously reported [20]. Fibroblasts were cultured in DMEM with 10% HI FBS and 1% antibiotic/antimycotic to 80% confluence in a 15-cm culture disk. After one rinse with PBS, the medium was changed to α-MEM containing 1000 mg/L d-glucose, 15% HI FBS, and 1% antibiotic–antimycotic. Upon reaching 100% confluence, the cells were washed twice with PBS then harvested using a cell scraper. The cells were then pelleted in PBS by centrifugation. Fibroblast pellets were lysed in 300 µL PBS with sonication and 400 µg of total glycoprotein was collected from the cell lysate. Free glycans were removed by Ultracel-10k Centrifugal filter (EMD Millipore, Billerica, Massachusetts) and the enriched glycoproteins were divided into 200-µg aliquots for *N*-glycan and *O*-glycan preparation. *N*-linked glycans were released from 200 µg of total glycoproteins or 10-µL plasma samples using PNGase F digestion (New England Biolabs, Ipswich, Massachusetts, USA). The released *N*-linked glycans from plasma or cells were purified and desalted by solid-phase extraction using a SepPak C18 and carbograph column. *O*-linked glycans were released using β-elimination with sodium borohydride. *O*-linked glycans from plasma or cells were purified and desalted using an AG 50 W-X8 resin cation exchange column. *N*-linked glycans or *O*-linked glycans were permethylated with sodium hydroxide and iodomethane in dimethyl sulfoxide (DMSO) as described before [21]. Following permethylation, glycans were extracted with water/chloroform (2:1, vol/vol) for four times and dried. Samples were then dissolved in 50% methanol, spotted with 11% 2,5-dihydroxylbenzoic acid matrix (1:1 vol/vol), and measured by MALDI-TOF using the positive mode on Ultraflex MALDI-TOF/TOF system (BrunkerDaltonics, Billerica, Massachusetts).

### Nucleotide sugar analysis

Fibroblasts were cultured in growth medium in 15-cm culture disks until 90–100% confluent, rinsed with PBS and harvested by scraping into PBS. Cells were counted using an automated cell counter (BioRad TC20) and pelleted at 500 RCF for 5 min. Nucleotide sugars were extracted based on previously published methods [22, 23]. Briefly, cell pellets were lysed in 70% ethanol, 300 µL of ethanol per million cells, and subjected to three rounds of sonication for 5 s at 15% amplitude. An aliquot of lysate was taken for total protein quantification. Lysates were pelleted at 16,000×*g* for 10 min at 4 °C and the supernatant was collected and lyophilized. The lyophilized supernatant was resuspended in 40 mM sodium phosphate buffer (pH 9.2) at 60 µL per million cells and further diluted based on total protein from the initial lysate relative to adult *WT*. The supernatant was applied to a Millipore spin column (10kD MW cut off) and spun for 30 min at 16,000×*g*. The extracted nucleotide sugars were analyzed by high-performance anion-exchange chromatography with pulsed amperometric detection (HPAEC-PAD) on a Dionex instrument as previously reported [22] and were quantified by comparison to a standard curve of dilutions of CMP-sialic acid (C8271, Sigma-Aldrich) in 40 mM sodium phosphate.

## Results

### Clinical report

**Fig. 1:**
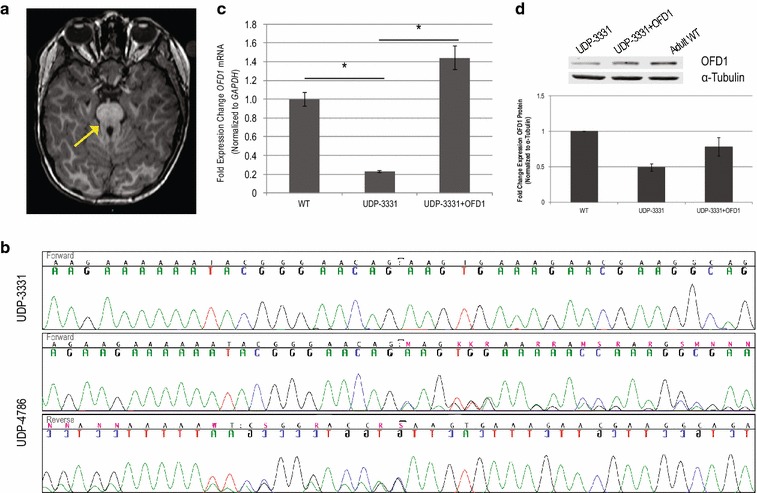
Clinical and molecular evidence for Joubert syndrome type 10 diagnosis. **a** Magnetic resonance imaging of the proband, UDP-3331, reveals the hallmark “molar tooth sign” of the superior cerebellar peduncles (*arrow*). **b** Sanger sequencing of both the proband, UDP-3331, and his mother, UDP-4786, confirms an *OFD1* single-nucleotide deletion (c.2656del) in the proband and maternal inheritance. **c** Analysis of mRNA transcripts from adult control cells (“WT”), UDP-3331, and UDP-3331 + OFD1 rescue shows reduced mRNA levels in the proband. *Error bars* represent standard error of four replicates; data are normalized to GAPDH expression and plotted relative to WT *OFD1* expression levels. *Asterisk* indicates *p* < 0.001 for two-tailed, heteroscedastic Student’s *t* test. **d** Western blot analysis of OFD1 levels in UDP-3331, UDP-3331 + OFD1 and adult WT primary fibroblast lines; one representative blot with OFD1 and α-tubulin is shown in the *top panels*; average of 3 replicate blots with independent lysates is shown in the *lower panel*. OFD1 levels are normalized to α-tubulin and plotted relative to WT OFD1 protein levels

The proband, UDP-3331, is a 5-year-old male, born to unaffected parents; he had an unaffected sister. At 14 months, he presented with severe feeding problems requiring placement of a G-tube. At 18 months, he was evaluated for hypotonia and dysmorphia. Over the next 4 years, his documented features included oral motor dysfunction, sleep apnea, myopathy, developmental delay, easy bruising, hypertrichosis, rocker bottom feet, and dysmorphic facial features, including a notched upper lip and submucous cleft palate. Following 5 years of non-diagnostic evaluations, the proband was evaluated at the NIH UDP. His brain MRI revealed a molar tooth sign (Fig. 1a) that, combined with his clinical features (Table 1), suggested a diagnosis of Joubert syndrome.

**Table 1:** Phenotype of JBTS10 patients

Joubert syndrome phenotypes (JBST1; OMIM: 213,300)		UDP-3331	NIH-452
Head and neck	Macrocephaly	+	+
Face	Prominent forehead	−	+
	High, rounded eyebrows	−	+
	Hemifacial spasms	−	−
	Low-set ears	+	+
	‘Tilted’ ears	−	−
Eyes	Abnormal, jerky eye movements	−	+
	Impaired smooth pursuit	+	+
	Impaired saccades	+	+
	Oculomotor apraxia	+	+
	Coloboma of optic nerve	−	−
	Chorioretinal coloboma	−	−
	Epicanthal folds	+	−
	Ptosis	−	+
Nose	Upturned nose	+	+
	Anteverted nostrils	−	+
Mouth	Triangular-shaped open mouth	+	+
	Protruding tongue	−	+
	Rhythmic tongue movements	−	−
Respiratory	Neonatal breathing dysregulation	+	+
	Hyperpnea, episodic	−	+
	Tachypnea, episodic	−	+
	Central apnea	+	+
Liver	Hepatic fibrosis (less common)	−	+
Central nervous system	Delayed psychomotor development	+	+
	Mental retardation/intellectual disability	+	+
	Ataxia	−	+
	Hypotonia	+	+
	Occipital meningocele (less common)	+	−
	Hypoplasia of the brainstem	−	−
	Malformation of brainstem structures	+	+
	‘Molar tooth sign’ on MRI	+	+
	Cerebellar vermis hypoplasia	+	+
	Dysgenesis or agenesis of the cerebellar vermis	+	+
	Deep posterior interpeduncular fossa	+	+
	Thick and elongated superior cerebellar peduncles	+	+
Behavioral psychiatric manifestations	Hyperactivity	−	−
	Aggressiveness	−	−
	Self-mutilation	+	+
Additional phenotypes	Submucosal cleft palate	+	−
	Notched upper lip or tongue	+	+
	Oral motor dysfunction	+	+
	Recurrent aspiration pneumonia	+	−
	Easy bruising	+	−
	Rockerbottom feet	+	−
	Hypertricosis	+	−
	Moderate hearing loss	+	−
	Double hair whorl	+	−
	GI tube placed in infancy	+	+
	GER (gastroesophageal reflux)	+	+
	Telecanthus	+	+
	Eczema	+	−
	Hip dysplasia	−	+
	Micropenis	−	+
	Pectus excavatum	−	+
	Growth retardation	−	+
	Elbow dislocation, chronic	−	+

Comparison of the standard Joubert syndrome features with those reported for UDP-3331 and NIH-452. Additional features noted in the patients are listed

### Exome sequencing identifies an *OFD1* mutation

Analysis of exome sequence data for variants in genes associated with Joubert syndrome revealed NM_003611.2: c.2656del, a maternally inherited hemizygous mutation in *OFD1*. Sanger sequencing confirmed the variant in the proband and mother (UDP-3331 and UDP-4786, respectively; Fig. 1b) and refined the diagnosis to JBTS10. Considering the apparently normal phenotype of the mother, no further testing or analysis of samples from individual UDP-4786 was performed.

### The OFD1 mutant protein is stable and appropriately localized

NM_003611.2: c.2656del, which occurred in the 20th exon, is predicted to encode a frameshift leading to a premature stop codon, p.Gln886Lysfs*2. To test if this mutation caused nonsense-mediated mRNA decay and thereby loss of OFD1 function, we measured *OFD1* mRNA levels in cultured dermal fibroblasts by qRT-PCR. Following normalization to *GAPDH* mRNA abundance, the UDP-3331 *OFD1* mRNA levels were approximately 30% of those in control dermal fibroblasts (Fig. 1c), a finding consistent with loss of function and previous reports [11]. OFD1 protein levels, as measured by immunoblotting, were approximately 50% of those in control dermal fibroblasts following normalization to α-tubulin (Fig. 1d). Lentiviral transduction of a WT copy of *OFD1* cDNA into the dermal fibroblasts of UDP-3331 partially rescued *OFD1* mRNA and protein expression (Fig. 1c, d).

**Fig. 2:**
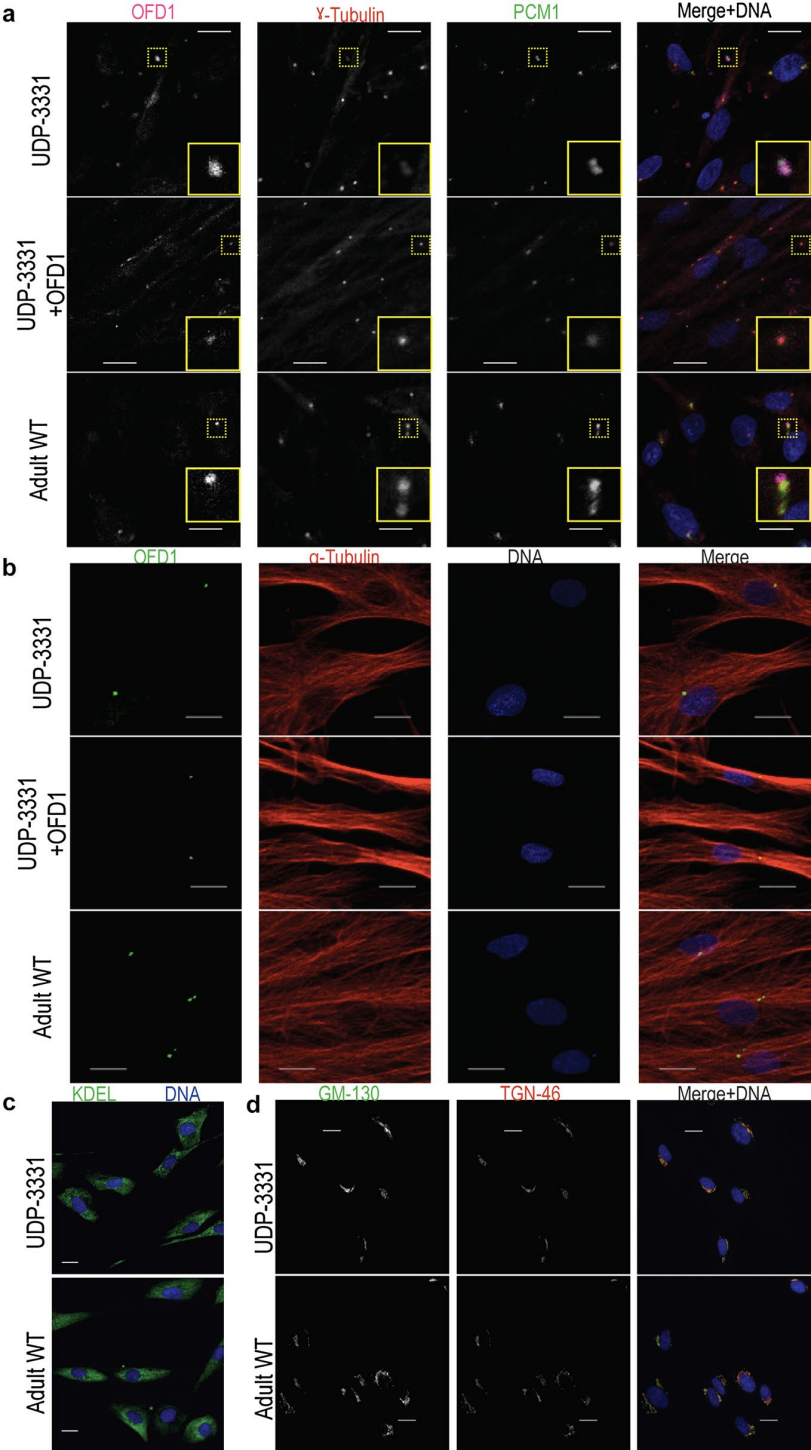
Cellular morphology and analysis of JBTS10 skin fibroblasts. **a** Co-localization analysis of OFD1 (*magenta*) signals in proband, proband rescued, and adult WT cells with ɣ-tubulin (*red*) and PCM1 (*green*). *Inset* higher magnification of the region outlined with *dotted lines*. **b** Immunofluorescence staining for OFD1 (*green*) and α-tubulin (*red*) in fibroblasts of the proband (UDP-3331), proband rescued (UDP-3331 + OFD1), and adult WT control. **c** Comparison of endoplasmic reticulum structure (KDEL, *green*) in UDP-3331 and WT cells. **d** Comparison of the *cis*- (GM-130, *green*) and *trans*- (TGN-46, *red*) Golgi in UDP-3331 and WT cells. DNA was stained with Hoechst 33,342 (*blue*). All *scale bars* represent 20 µm

We next determined the intracellular localization of the residual OFD1 protein by immunofluorescent studies of dermal fibroblasts. In both UDP-3331 and control, residual OFD1 localized with the centrosome as judged by co-localization with centrosome markers ɣ-tubulin and PCM1 (Fig. 2a). Notably, in both affected and control fibroblasts, we observed variable co-localization of all three centrosomal proteins; the variability is likely due to the presence of non-centrosomal aggregates of PCM1 [24] and cell-cycle differences among the population of cells resulting in variable ɣ-tubulin localization to the centrosome [25, 26]. Microtubule morphology (α-tubulin) appeared to be unaffected in the JBTS10 cell line as compared to control (Fig. 2b).

Given the role of the basal body and microtubules in regulating trafficking through the secretory pathway, we also examined the structure of these compartments in the affected cells [27]. As judged by immunofluorescence, there was no change in the morphology of the endoplasmic reticulum and the *cis*- and *trans*-Golgi of UDP-3331 compared to control adult dermal fibroblasts (Fig. 2c, d).

### Decreased cilium number in *OFD1* p.Gln886Lysfs*2 mutant cells

**Fig. 3:**
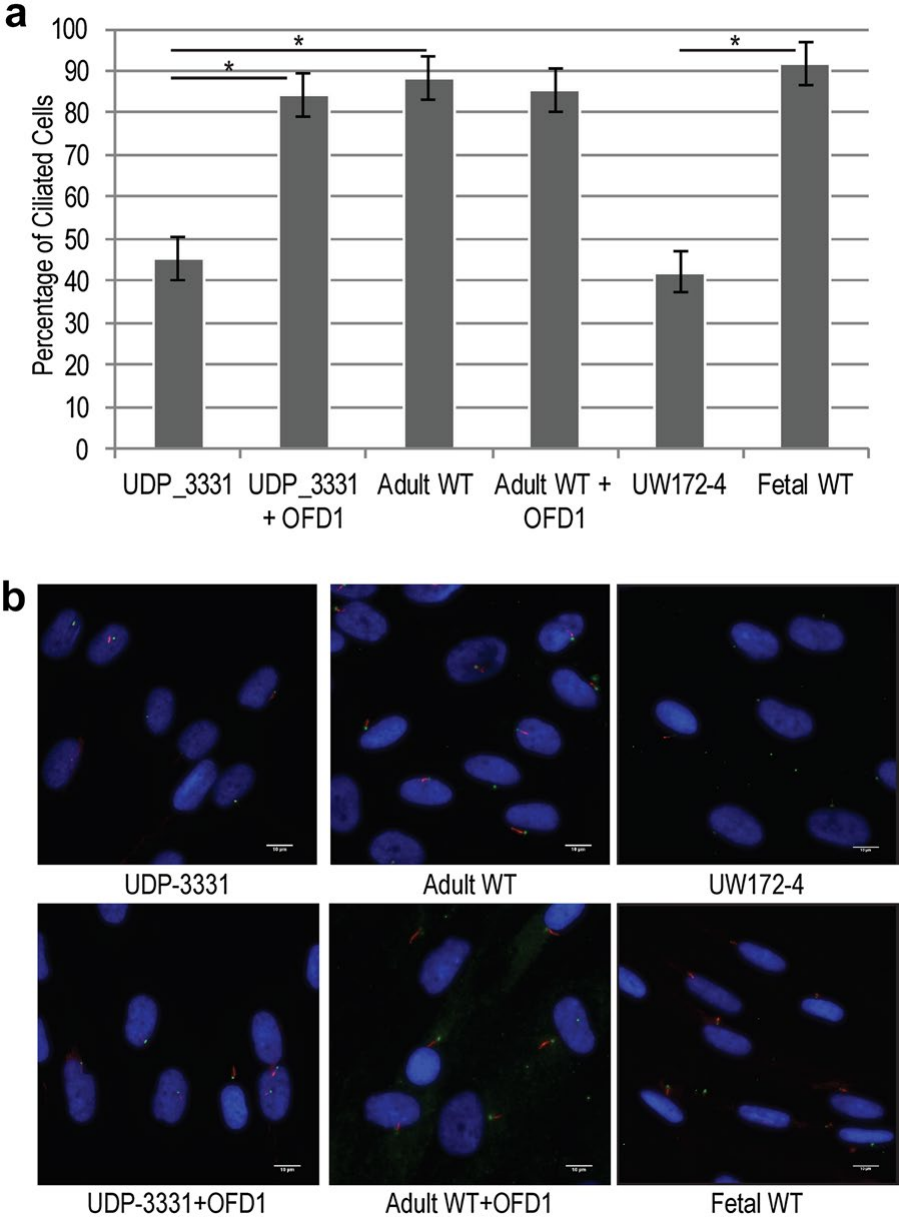
Analysis of ciliogenesis in JBTS10 and control skin fibroblasts. **a** The percentage of ciliated cells following 48 h of serum starvation in UDP-3331, UDP-3331 + OFD1 rescue, adult WT, adult WT + OFD1 overexpression, UW172-4, and fetal WT control cells. *Error bars* represent the 95% confidence interval for the average of three independent experiments. *Asterisks* indicate *p* < 0.001, Student’s *t* test. **b** Representative images of ciliated cells for the indicated *cell lines*. Acetylated tubulin (*red*) and ɣ-tubulin (*green*) mark the cilia and basal body, respectively. DNA was stained with DAPI (*blue*). *Scale bars* 10 µm

The above observations raised the question as to whether the remaining OFD1 protein that localized to the centrosome in UDP-3331 dermal fibroblasts was sufficient to support ciliogenesis. Comparable to a fetal-affected JBTS10 cell line (UW172-4, NM_003611.2: c.277G > T, p.Val93Phe [28]), UDP-3331 dermal fibroblasts showed a decreased ability to form cilia that was rescued to near WT levels by expression of WT *OFD1* (Fig. 3a, b). Thus, consistent with previous studies [8], the reduction in OFD1 mRNA and protein levels in UDP-3331 dermal fibroblasts is sufficient to impede cilia formation.

### JBTS10 tissues have OFD1-dependent anomalies of protein glycosylation

Evaluation of UDP-3331 included a screen for anomalies of *N*- and *O*-linked glycans in plasma and of free oligosaccharides in urine. This detected a decrease in mature, fully sialylated and galactosylated *N*-linked glycans at *m/z* 2973 (Table 2), whereas monosialo glycan at *m/z* 2431 and monosialo, monogalacto glycan at *m/z* 2227 were increased. In plasma *O*-linked glycans sialylated T antigen (see Additional file 1: Table S1) was decreased, and T antigen was at the low end of the normal range. Both the plasma *N*- and *O*- glycosylation profiles indicated an abnormality in sialic acid utilization in the *trans*-Golgi or abnormal metabolism of sialic acids (see Additional file 2: Figure S1). Urinary oligosaccharides were within the normal range. Supporting the association of glycosylation anomalies with JBTS10, similar plasma *N*-linked findings were detected in another individual with JBTS10 (NIH-452, NM_003611.2: c.149A>G [29, 30], Table 2); *O*-linked glycosylation was not tested in this individual due to limited sample amount.

**Table 2:** Summary of *N*-glycan profiles of JBTS10 patient plasma

Centroid mass (*m/z*)	Predicted glycan species	UDP-3331 (% total glycans)	NIH-452 (% total glycans)	Ref low (% total glycans)	Ref high (% total glycans)
1580.2	Hex5 HexNAc2	0.69	0.91	0.23	1.88
1662.3	Hex3 HexNAc4	0.03	0.00	0.00	0.22
1784.4	Hex6 HexNAc2	0.51	0.48	0.00	0.87
1866.5	Hex4 HexNAc4	0.19	0.00	0.06	0.53
1982.5	Hex4 NeuAc1 HexNAc3	0.48	0.43	0.08	0.80
1988.5	Hex7 HexNAc2	0.19	0.00	0.00	0.39
2070.6	Hex5 HexNAc4	–	0.15	0.00	0.30
2156.7^#^	Hex4 dHex1 HexNAc3 NeuAc1	0.05	0.00	0.06	0.17
2186.7	Hex4 dHex1 NeuGc1 HexNAc3	–	0.39	0.00	0.89
2192.7	Hex8 HexNAc2	0.32	0.00	0.06	0.61
2227.7^^^	Hex4 HexNAc4 NeuAc1	1.38	1.54	0.40	1.28
2285.2^^^	Hex4 dHex1 HexNAc5	0.17	–	0.00	0.16
2431.9^^^	Hex5 HexNAc4 NeuAc1	5.87	9.88	2.38	6.74
2606.0	Hex5 dHex1 HexNAc4 NeuAc1	0.54	1.87	0.79	2.87
2793.3^†^	Hex5 HexNAc4 NeuAc2	20.29	40.58	28.38	58.23
2966.5	Hex5 dHex1 HexNAc4 NeuAc2	1.49	2.89	2.49	3.36
3241.6	Hex6 HexNAc5 NeuAc2	1.13	1.52	0.08	1.70
3415.6	Hex6 dHex1 HexNAc5 NeuAc2	0.25	0.00	0.00	0.87
3602.9	Hex6 HexNAc5 NeuAc3	3.31	6.72	1.64	6.10
3776.6^#^	Hex6 dHex1 HexNAc5 NeuAc3	0.94	0.38	0.00	7.27

The percentages of total glycans for the indicated *N*-glycan species in the plasma of UDP-3331 and NIH-452 are shown along with reference (Ref) range derived from controls. Species with values in the low-normal range in the JBTS10 samples are flagged with a pound sign (#). Species below the normal range in JBTS10 sample(s) are flagged with a dagger (†). Species that are elevated above the normal range are flagged with a caret (^)

^#^Low normal in JBST10

^^^Above normal in JBST10

^†^Below normal in JBST10

To exclude the possibility that these findings were nonspecific, we defined the *N*- and *O*-linked glycome profile in cultured dermal fibroblasts from JBTS10 patients UDP-3331 and UW172-4. Both cell lines had increased levels of mono-sialylated *N*-glycan species compared to controls (Table 3). Both cell lines also had abnormal formation of core 2 mature, sialylated *O*-linked glycans (see Additional file 3: Table S2) and thus a relative high abundance of the core 1 species. Core 1 sialylated T antigen from cellular protein was not reduced. Each *N*-glycosylation abnormality in the cultured JBTS10 dermal fibroblasts was partially rescued by expression of WT OFD1 (Table 3, UDP-3331 + OFD1). As was observed for the plasma results in Table 2, we observed a defect in sialylated glycans in JBTS10 patient fibroblast samples.

**Table 3:** Summary of *N*-glycan profiles of JBTS10 patient fibroblasts

Centroid mass (*m/z*)	Glycan structure	UDP-3331 (% total glycans)	UDP-3331 +OFD1 (% total glycans)	UW172–4 (% total glycans)	Adult control (% total glycans)	Ref low (% total glycans)	Ref high (% total glycans)
1171.9	Hex3 HexNAc2	4.09	7.07	1.63	5.00	0.23	12.38
1346.0	Hex3 dHex1 HexNAc2	3.86	5.28	1.06	5.09	0.00	6.11
1417.1^#^	Hex3 HexNAc3	0.00	0.79	0.00	0.78	0.00	0.78
1580.2	Hex5 HexNAc2	8.19	11.68	4.55	8.46	1.83	14.29
1621.3	Hex4 HexNAc3	0.56	0.76	0.00	0.98	0.00	0.98
1662.3	Hex3 HexNAc4	0.00	0.00	0.00	0.00	0.00	0.29
1784.4	Hex6 HexNAc2	19.88	17.97	15.17	18.31	6.54	20.74
1825.4	Hex5 HexNAc3	0.57	0.62	0.00	0.90	0.00	0.90
1866.5	Hex4 HexNAc4	0.74	0.81	0.00	0.66	0.00	0.70
1982.5	Hex4 NeuAc1 HexNAc3	0.00	0.00	0.00	0.00	0.00	0.30
1988.5	Hex7 HexNAc2	9.81	9.62	12.04	9.67	4.87	14.86
2029.6	Hex6 HexNAc3	0.54	0.52	0.00	0.93	0.00	2.19
2070.6	Hex5 HexNAc4	4.39	4.03	2.03	3.20	0.00	3.88
2156.7^#^	Hex4 dHex1 HexNAc3 NeuAc1	0.00	0.34	0.00	0.52	0.00	0.75
2192.7	Hex8 HexNAc2	16.18	13.96	27.66	16.04	0.00	32.90
2227.7	Hex4 HexNAc4 NeuAc1	0.00	0.00	0.00	0.00	0.00	0.20
2396.9	Hex9 HexNAc2	9.28	7.49	18.11	9.09	0.24	32.34
2431.9^^^	Hex5 HexNAc4 NeuAc1	2.18	1.47	2.92	1.83	0.00	1.83
2606.0^^^	Hex5 dHex1 HexNAc4 NeuAc1	6.85	4.19	2.23	4.70	0.00	4.70
2793.3	Hex5 HexNAc4 NeuAc2	0.86	0.45	1.53	1.00	1.21	6.80
2967.3	Hex5 dHex1 HexNAc4 NeuAc2	2.69	1.45	4.74	2.02	3.38	10.79
3241.6	Hex6 HexNAc5 NeuAc2	0.00	0.00	0.00	0.17	0.00	1.96
3415.6	Hex6 dHex1 HexNAc5 NeuAc2	0.45	0.22	0.84	0.39	0.43	5.84
3602.9^†^	Hex6 HexNAc5 NeuAc3	0.33	0.00	0.00	0.28	0.64	23.23
3776.6^#^	Hex6 dHex1 HexNAc5 NeuAc3	0.21	0.00	0.79	0.11	0.00	5.46

The percentages of total glycans of the indicated *N*-glycan species for UDP-3331, UDP-3331 + OFD1 rescue, UW172-4, and adult WT control fibroblasts samples are shown along with reference (Ref) low and high values derived from control samples. Species with values in the low-normal range in the JBTS10 sample(s) are flagged with a pound sign (#). Species that are elevated above the normal range in JBTS10 sample(s) are flagged with a caret (^). Species below the normal range in JBTS10 sample(s) are flagged with a dagger (†)

^#^Low normal in JBST10

^^^Above normal in JBST10

^†^Below normal in JBST10

### Cultured JBTS10 dermal fibroblasts have increased nuclear CMP-sialic acid

**Fig. 4:**
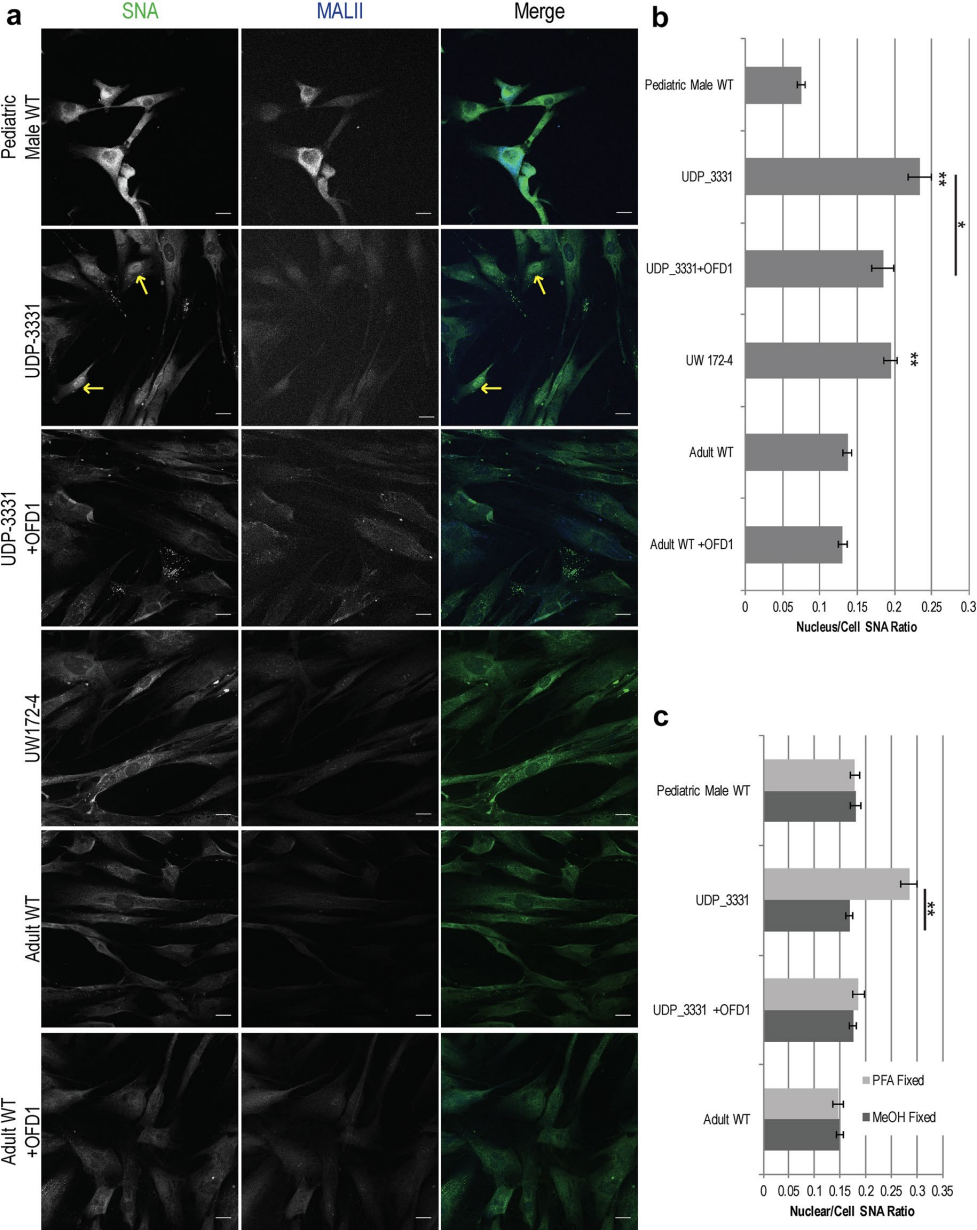
Lectin staining for sialic acid epitopes in JBTS10 and control skin fibroblasts. **a** Lectin staining of α-2,6-sialic acid (SNA, *green*) and α-2,3-sialic acid (MALII, *blue*) linkages in affected, rescued and control cells, as indicated. *Arrows* indicate atypical nuclear staining by SNA in UDP-3331. *Scale bars* 20 µm. **b** Quantification of the fluorescence signal for SNA in nucleus relative to the entire cell for affected, rescued, and control cells under paraformaldehyde fixation conditions. *Error bars* represent standard error. *Single asterisks* indicate *p* < 0.05; *double asterisks* indicate *p* < 0.001 relative to both adult and pediatric male controls (unpaired, heteroscedastic *t* test). **c** Comparison of methanol and paraformaldehyde fixation on the relative SNA staining intensity of the nucleus vs. the whole cell. *Double asterisks* indicate *p* < 0.001(unpaired, heteroscedastic *t* test). *Error bars* represent standard error

To characterize further the glycomic profile abnormalities, we performed lectin immunofluorescence using SNA, which preferentially binds to α-2,6-linked sialic acids, and MALII, which primarily binds α-2,3-linked sialic acids. We observed increased nuclear localization of the SNA fluorescently labeled lectin signal in both of the JBTS10 dermal fibroblast lines, and this was reduced upon rescue with WT OFD1 (Fig. 4a, b, *p* < 0.05).

**Fig. 5:**
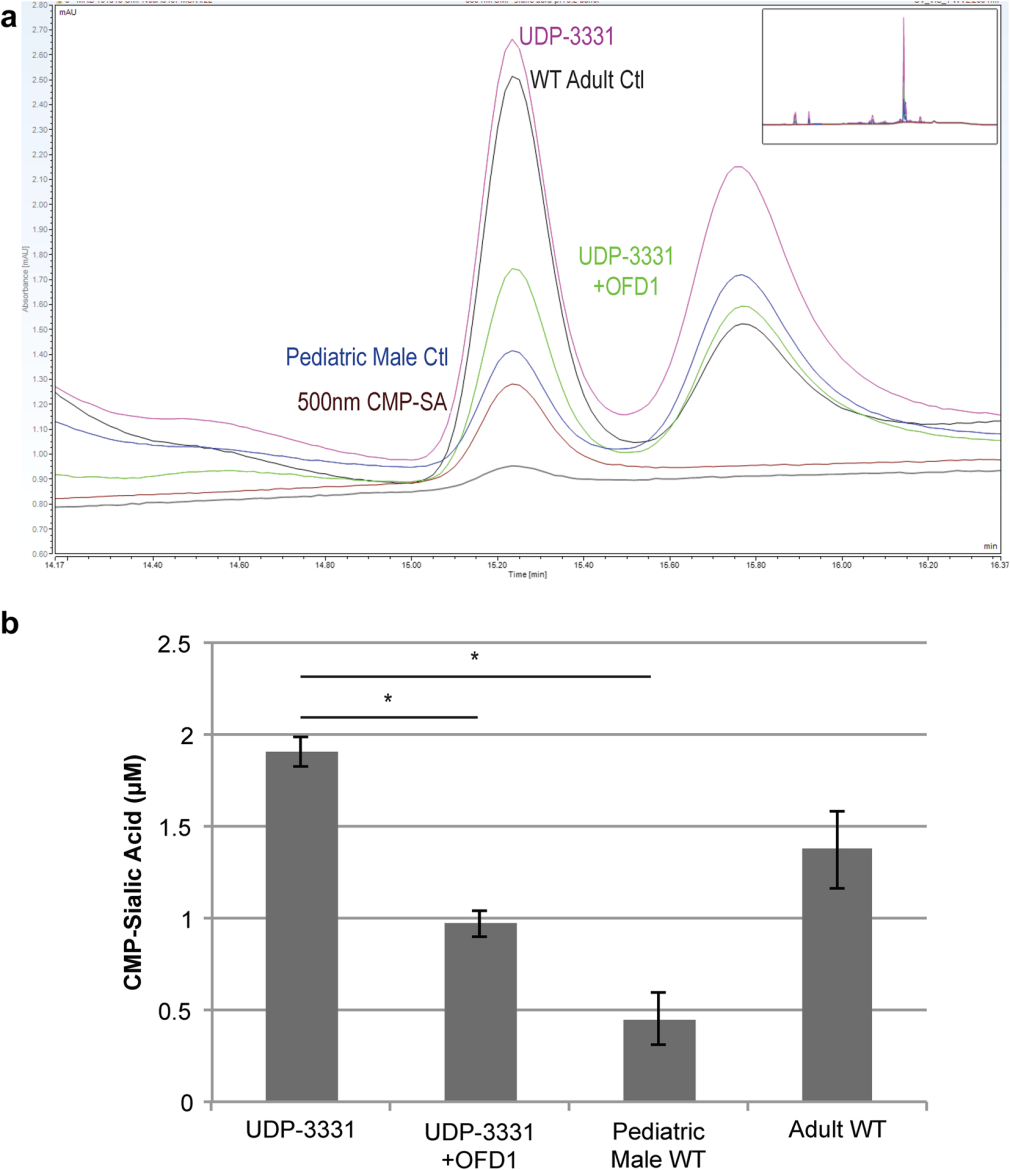
Nucleotide sugar analysis in JBTS10 and control skin fibroblasts. **a** Representative HPAEC trace for UDP-3331, UDP-3331 + OFD1 rescue, adult WT, adult WT + OFD1 overexpression, pediatric male control cells, and 500 nM standard CMP-sialic acid. **b** Quantification of CMP-sialic acid levels in affected and control cells (in μM) (*single asterisks* indicate p < 0.005 in an unpaired, heteroscedastic *t* test). *Error bars* represent standard error

Given that the increased nuclear SNA signal was only observed in JBTS10 cells fixed with paraformaldehyde but not with methanol (Fig. 4c), we hypothesized that the SNA-reactive epitope was a small molecule, lipid, or protein not preserved by methanol fixation. Unlike other nucleotide sugars, which are synthesized in the cytosol, CMP-sialic acid (CMP-SA) is synthesized in the nucleus and, under aldehyde fixation, could constitute a small SNA-reactive epitope [31]. Using HPAEC-PAD, comparison of nucleotide sugars levels between UDP-3331 and adult WT cultured dermal fibroblasts showed a 39% increase in CMP-SA in UDP-3331 (Fig. 5a, b); introduction of WT OFD1 decreased the CMP-SA levels by 50% (*p* < 0.005, unpaired *t* test).

### Neuraminidase treatment destabilizes cellular primary cilia

**Fig. 6:**
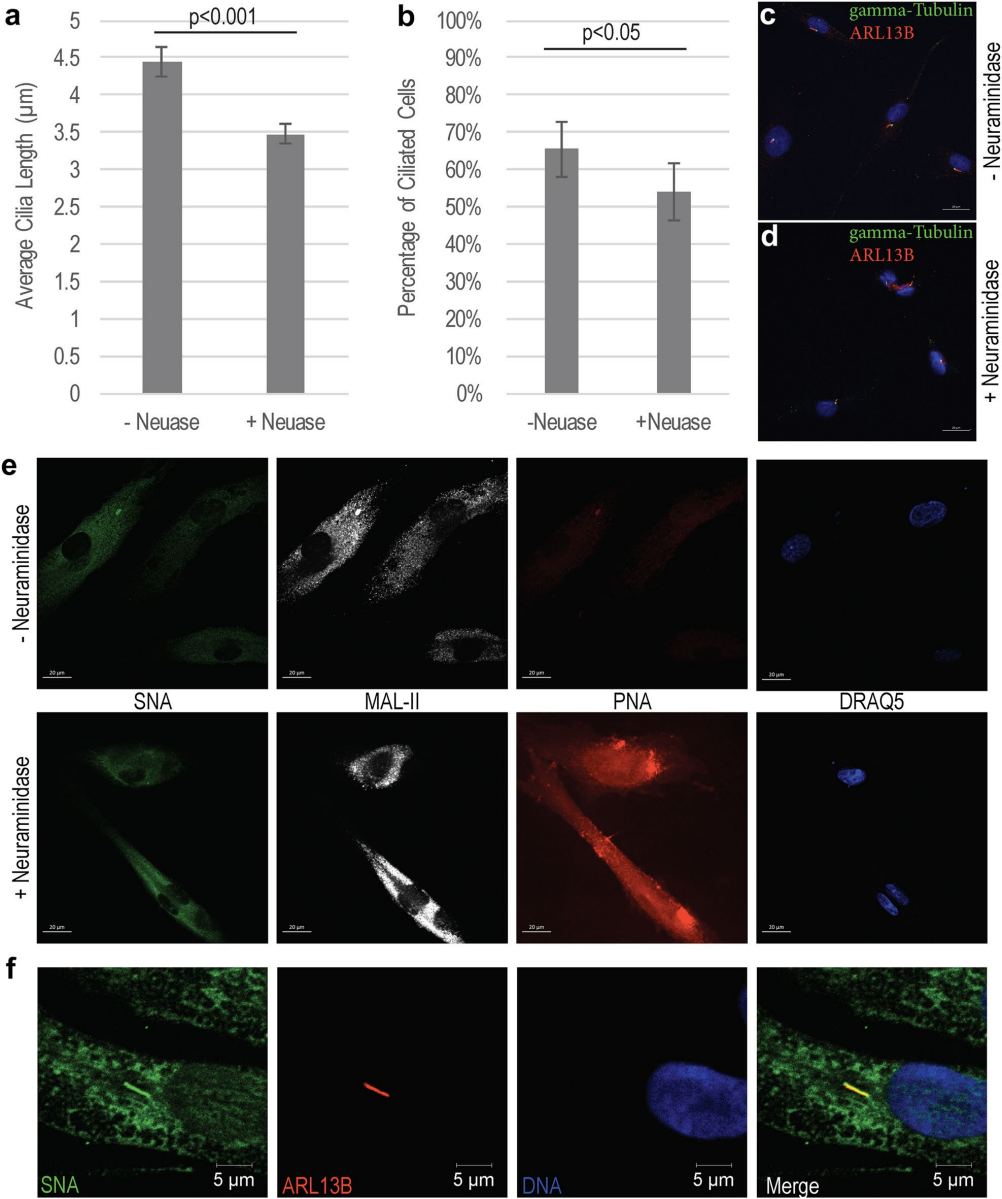
Neuraminidase treatment of adult WT primary cells. **a** Average cilia length of WT cells with and without neuraminidase (±Neuase) treatment to remove sialic acids from glycan chains; neuraminidase treatment significantly reduced average cilia length by ~1 µm. *Error bars* represent standard error; *p* < 0.001 as determined in an unpaired, heteroscedastic *t* test. **b** The percentage of ciliated cells is decreased after neuraminidase treatment; *p* < 0.05 as determined by a two-tailed Fisher’s Exact *t* test; *error bars* represent the 95% confidence interval for the binomial distribution represent by the sample population. **c**, **d** Representative images of WT fibroblasts without (**c**) and with (**d**) neuraminidase treatment. Cilia are stained with ARL13B antibody (*red*) and ɣ-tubulin (*green*). *Scale bars* 20 µm. **e** Lectin staining for sialic acid with SNA (*green*) and MAL II (*white*) and galactose with PNA (*red*) in WT fibroblasts with DRAQ5 DNA counterstaining (*blue*). **f** Representative image of SNA (*green*) signal showing alpha 2,6-sialylation co-localizing with ARL13B-positive cilia (*red*) in WT cells without neuraminidase treatment. *Scale bars* 5 µm

Given the association between abnormal sialylation and ciliogenesis in the JBTS10 patient lines, we sought to examine the effects of disrupting sialylation on cilia stability in control cells. Following growth in ciliogenic conditions, WT cells were either left untreated or treated with neuraminidase to remove sialic acids from the cellular glycoproteome. The neuraminidase used is isolated from *Clostridium perfringens* and can cleave α-2,3-, α-2,6-, or α-2,8-linked sialic acids, although it has highest affinity for α-2,3-linkages. Cells were then fixed and stained for cilia markers and cilia were measured. We observed a significant decrease (*p* < 0.001) in average cilia length in WT cells following neuraminidase treatment and a modest (*p* < 0.05) decrease in the number of ciliated cells in the population (Fig. 6a–d). Neuraminidase treatment successfully disrupted glycoprotein sialylation as indicated by increased lectin staining with PNA, which strongly binds galactose epitopes normally masked by terminal sialic acids (Fig. 6e). Additionally, in the absence of neuraminidase treatment, we observed co-localization of SNA-reactive epitopes with the cilium marker ARL13B, indicating that some proteins localized to the primary cilia are sialylated (Fig. 6f).

## Discussion

Herein we have detailed the clinical and molecular phenotypes of a boy, UDP-3331, with Joubert syndrome type 10 (JBTS10). His clinical, molecular, and cellular findings are consistent with other reports of JBTS10. Interestingly, we observed a global alteration in the *N*- and *O*-linked glycosylation profiles of three JBTS10 patients. The most pronounced and consistent glycosylation change was a decrease of mature, fully sialylated *N*-glycans and an increase in total cellular CMP-sialic acid. Within cultured skin fibroblasts, lentiviral rescue with WT *OFD1* cDNA normalized the abnormal glycosylation profiles in JBTS10 patient cells and the ciliogenesis phenotype previously associated with *OFD1* mutations.

The observed decrease in primary cilia stability following neuraminidase removal of sialic acids from the glycoproteome and the co-localization of sialylated glycoproteins with the primary cilium suggest that protein sialylation plays a role in ciliary homeostasis. We hypothesize, therefore, that the abnormal glycosylation might contribute to the ciliogensis defect. Two factors, however, remain to be addressed. First, our glycomic studies on primary dermal fibroblasts were performed under growth conditions that do not promote ciliogenesis, and therefore, it is unclear how much of the abnormal glycosylation is attributable to ciliary proteins. Second, because of technical limitations to performing glycome profiling on the cilia, we are unable to assess biochemically whether the ciliary proteome is aberrantly glycosylated in patient cells. These two limitations provide testable hypotheses for future investigations.

Regardless of the impact directly on the ciliary proteins, the accumulation of the sialic acid epitope that binds to the lectin SNA in the nuclei of JBTS10 patient cells and the increased CMP-SA and the accumulation of incompletely sialylated *N*-glycans are consistent with a defect in CMP-SA metabolism or transport. Unlike other nucleotide sugars, CMP-SA is synthesized in the nucleus, rather than the cytosol; thus, CMP-SA must be transported through two distinct membrane-bound organelles for proper sialylation to occur [32, 33]. There are many possible ways in which alterations in levels of functional OFD1 could lead to changes in sialylated glycoproteins. First, nuclear OFD1 [13] might contribute to CMP-SA metabolism. Second, defects of ciliogenesis might alter cell signaling [34–36] or the role of the basal body in regulating microtubules and intracellular transport to impair CMP-SA transport from the nucleus and subsequent utilization in the Golgi. Disruption of the centrosome structure and tethering of the daughter centrioles are associated with abnormal Golgi structure [37], and alterations in the structure of the Golgi cisternae would undoubtedly influence the glycosylation enzymes that are targeted specifically to *cis*- or *trans*-Golgi compartments [38]. However, we did not observe any obvious changes in centriole separation or Golgi structure. Third, the increased sialylation of *O*-linked core 1 T antigen on cellular proteins and the decreased sialylation of *O*-linked core 1 T antigen on secreted glycoproteins in plasma might suggest altered exocytosis and endocytosis of glycoproteins. Finally, elevated processing of sialoglycoproteins or enhanced turnover of surface sialoglycoproteins might contribute to the abnormal glycosylation signature observed [39]. Each of these potential mechanisms highlights the important role of ciliary and basal body proteins in cell physiology and the delicate homeostatic balance that can be indirectly disrupted by single-gene mutations.

Prior associations between primary ciliopathies and altered glycosylation have been limited to observations of the mutated ciliary proteins. Polycystin-2, polycystin-1, and aquaporin-11 mutations associate with abnormal glycosylation of ciliary proteins [40–42]. Changes in protein trafficking, which result in altered protein glycosylation, have been linked to polycystic liver disease [43].

There exists corroborating evidence that altered glycosylation might contribute to the pathogenesis of some ciliopathies. Primary defects in glycosylation, collectively referred to as Congenital Disorders of Glycosylation (CDGs), cause phenotypes similar to those of primary ciliopathies and disrupt cilia function. Defects in the *N*-linked glycosylation genes *ALG3, ALG8,* and *ALG9,* as well as the *O*-linked glycosylation gene *B3GALTL,* variably result in polycystic kidneys, cleft lip or palate, polycystic ovaries, and abnormal development of the brain including the corpus callosum [44–47]. Mutations in *GALNT11*, encoding polypeptide *N*-acetylgalactosaminyltransferase, cause ciliary dysfunction and heterotaxy [48].

The importance of glycosylation in neural development is well illustrated by animal models of disrupted glycosylation. A knock-out of the only sialyltransferase gene in *D. melanogaster* caused significant neurological symptoms, implicating sialylation as a key factor in neurodevelopment and function [49]. Additionally, disruption of proteoglycan formation in *C. elegans* by alteration of microRNA levels causes aberrant migration of neurons [50]. Alterations of polysialic acid epitope formation in mice also cause abnormal neuronal development and disrupted neuron migration, likely due to changes in sialylation of NCAM [51]. The cerebellum and midbrain anomalies that result in the molar tooth sign (MTS) are thought to arise due to aberrant migration of neurons in the cerebellum. Given that both *N*- and *O*-linked CDGs present with abnormal development of other brain structures, and animal models with altered sialylation show distinct neurodevelopment defects, it is possible that the altered glycosylation we observe in JBTS10 patients may contribute to the formation of MTS. Recent work by Feng and colleagues to identify the *N*-glycoproteome in the mouse brain revealed that several key proteins for brain development are *N*-glycosylated [52].

## Conclusions

Our findings highlight a potential role for glycosylation in contributing to pathogenesis of JBST10 and possibly other ciliary disorders. The mechanism for the altered glycosylation remains to be determined. Our observations raise the question as to whether glycosylation is altered in other types of Joubert syndrome and more severe ciliopathies. The impact of altered glycosylation in contributing to pathophysiology in ciliopathies bears further study.

## Additional files

**Additional file 1: Table S1.** *O-*glycan analysis results of UDP-3331 plasma.

**Additional file 2: Figure S1.** Overview of *N-* and *O*-linked glycosylation pathways. *N*-linked glycosylation begins with the synthesis of the dolichol phosphate-linked glycan precursor on the cytosolic face of the endoplasmic reticulum (E.R.). Once the precursor molecule is flipped into the E.R. lumen, it is further built up before being transferred to the nascent polypeptide. After the glycosylated polypeptide is properly folded, it is transferred to the *cis* Golgi where the high-37 mannose species are trimmed and further matured by the addition *N*-acetylglucosamine (GlcNAc), followed by galactose, fucose and, finally, sialic acid to form the most prevalent, Complex *N*-linked glycans. An alternative pathway to generate Hybrid glycan structures also exists. The enzymes that perform each of these glycan-building steps are targeted to either *cis, medial*, or *trans* Golgi stacks to facilitate proper assembly of the sugars. Two representative *O*-linked glycosylation pathways are shown: *O*-GalNAc and *O*-mannose. Much of the *O*-linked glycosylation reactions occur in the Golgi apparatus and, similar to *N*-linked glycosylation, the complexity and length of the sugars extends as the protein transits through the Golgi cisternae. In JBTS10 patient samples, we observe a decrease in formation of the mature, fully sialiylated *N-*glycans and alterations in levels of the *O*-linked sialylated species. These species are shown in *trans* Golgi cisternae, which has been highlighted in blue.

**Additional file 3: Table S2.** *O-*glycan analysis results of UDP-3331 and UW172-4 primary dermal fibroblasts.

## Abbreviations

JBTS10: Joubert syndrome type 10
JBTS: Joubert syndrome
OFDI: orofaciodigital syndrome I
SGBS2: Simpson–Golabi–Behmel syndrome, type 2
PCP: planar cell polarity
NIH UDP: National Institutes of Health Undiagnosed Diseases Program
DMEM: Dulbecco’s Modified Eagle Medium
HI FBS: Heat inactivated fetal bovine serum
PBS: phosphate-buffered saline
WT: wild-type
HPAEC-PAD: high-performance anion-exchange chromatography with pulsed amperometric detection
CMP-SA: CMP-sialic acid
CDG: congenital disorder of glycosylation
NHGRI: National Human Genome Research Institute
