# Supplementary material for: Abnormal glycosylation in Joubert syndrome type 10
Source: Cilia. 2017 Mar 23;6:2. doi: 10.1186/s13630-017-0048-6 (PMC5364566; doi:10.1186/s13630-017-0048-6)
Supplement: Supplementary file 1 — Additional file 1: Table S1. O-glycan analysis results of UDP-3331 plasma. [file 13630_2017_48_MOESM1_ESM.docx]

Table S1 Plasma *O*-Glycan analysis of UDP-3331

| Glycan Composition |  | | | UDP-3331 (µmol/L) | Normal Low (µmol/L) | Normal High (µmol/L) |
| --- | --- | --- | --- | --- | --- | --- |
| T antigen | |  | 0.37 | | 0.22 | 1.14 |
| **Sialyl-T antigen** | | **†** | 11.0 | | 11.7 | 31.4 |
| T antigen/Sialyl-T antigen | |  | 0.03 | | 0.00 | 0.06 |

† Below normal in JBST10

Table S1: Plasma *O*-glycan analysis of UDP-3331

Analysis of plasma *O*-glycans in UDP-3331 shows low levels of the Sialyl-T antigen and borderline low levels of T antigen.
