# Supplementary figures and images for: Abnormal glycosylation in Joubert syndrome type 10
Source: Cilia. 2017 Mar 23;6:2. doi: 10.1186/s13630-017-0048-6 (PMC5364566; doi:10.1186/s13630-017-0048-6)

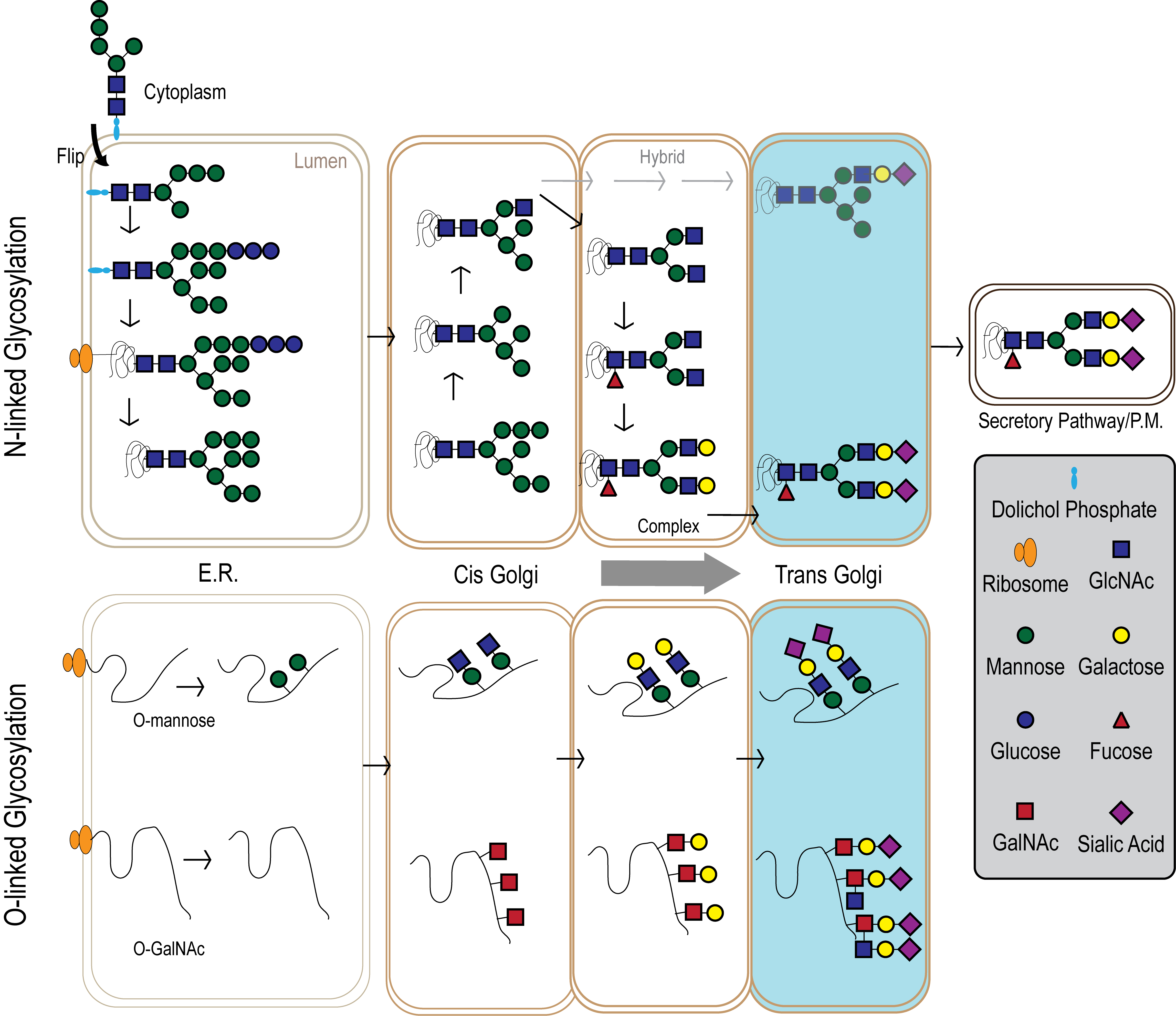

Supplement: Supplementary file 2 — Additional file 2: Figure S1. Overview of N- and O-linked glycosylation pathways. N-linked glycosylation begins with the synthesis of the dolichol phosphate-linked glycan precursor on the cytosolic face of the endoplasmic reticulum (E.R.). Once the precursor molecule is flipped into the E.R. lumen, it is further built up before being transferred to the nascent polypeptide. After the glycosylated polypeptide is properly folded, it is transferred to the cis Golgi where the high-37 mannose species are trimmed and further matured by the addition N-acetylglucosamine (GlcNAc), followed by galactose, fucose and, finally, sialic acid to form the most prevalent, Complex N-linked glycans. An alternative pathway to generate Hybrid glycan structures also exists. The enzymes that perform each of these glycan-building steps are targeted to either cis, medial, or trans Golgi stacks to facilitate proper assembly of the sugars. Two representative O-linked glycosylation pathways are shown: O-GalNAc and O-mannose. Much of the O-linked glycosylation reactions occur in the Golgi apparatus and, similar to N-linked glycosylation, the complexity and length of the sugars extends as the protein transits through the Golgi cisternae. In JBTS10 patient samples, we observe a decrease in formation of the mature, fully sialiylated N-glycans and alterations in levels of the O-linked sialylated species. These species are shown in trans Golgi cisternae, which has been highlighted in blue. [file 13630_2017_48_MOESM2_ESM.tif]
