# Supplementary material for: Abnormal glycosylation in Joubert syndrome type 10
Source: Cilia. 2017 Mar 23;6:2. doi: 10.1186/s13630-017-0048-6 (PMC5364566; doi:10.1186/s13630-017-0048-6)
Supplement: Supplementary file 3 — Additional file 3: Table S2. O-glycan analysis results of UDP-3331 and UW172-4 primary dermal fibroblasts. [file 13630_2017_48_MOESM3_ESM.docx]

Table S2: Fibroblast *O-*glycan profiles in JBTS10 cell lines

| Centroid Mass m/z | | Predicted Glycan Species | | UDP-3331 | UW  172-4 | Ref Low | | Ref High | |  |
| --- | --- | --- | --- | --- | --- | --- | --- | --- | --- | --- |
| 534.3 |  | core1 T antigen | | 0.00% | 4.24% | 1.42% | | 8.06% | |  |
| 708.3 |  | core1 T antigen fucosylated | | 7.85% | 0.00% | 0.00% | | 0.00% | |  |
| **895.4** | **^** | core 1 sialylated T O-glycan | | 14.78% | 17.00% | 6.20% | | 14.61% | |  |
| **983.5** | **#** | core2, asialylated Hexose3HexNAc2 | | 0.00% | 2.11% | 2.75% | | 14.65% | |  |
| 1157.8 |  | Fuc1 asialylated hexose3HexNAc2 core2 | | 0.00% | 0.00% | 0.00% | | 0.86% | |  |
| **1256.6** | **^** | core1, disialyl T | | 16.30% | 29.73% | 1.66% | | 12.23% | |  |
| 1331.7 |  | Fuc2Hexose2HexNAc2 | | 0.00% | 0.00% | 0.00% | | 0.00% | |  |
| **1344.6** | **†** | sial1hexose2HexNAc2 (Core 2) | | 0.00% | 9.22% | 15.76% | | 19.90% | |  |
| 1705.8 |  | core2, disialyl, sial2hexose2hexNAc2 | | 23.64% | 20.09% | 7.50% | | 32.87% | |  |
| **1794.2** | **#** | core2 extended, sial1hexose3HexNAc3 | | 0.00% | 0.00% | 0.00% | | 3.43% | |  |
| **2243.3** | **†** | core2, disialyl, sial2hexose3hexNAc3 | | 0.00% | 0.00% | 1.77% | | 4.61% | |  |
|  | | |  |  |  |  | |  | |  |
| **Core 1/Core 2 [(534+895+1256)/(983+1344+1706)]** ^ | | | | 1.32 | 1.62 | | 0.36 | | 0.52 | |
| **Sialylation of core 1 [534/895]** # | | | | 0.00 | 0.25 | | 0.23 | | 0.55 | |

#Low-normal in JBST10 ^Above normal in JBST10 † Below normal in JBST10

Table S2: Fibroblast *O-*glycan profiles in JBTS10 cell lines

The percent of total glycans of the indicated *O-*glycan species for UDP-3331 and UW172-4 are shown along with the reference (Ref) low and high values derived from control samples. The ratios of various core 1 *vs.* core 2 species are shown at the bottom of the table with the corresponding centroid mass values in brackets. Species with values in the low-normal range in the JBTS10 sample(s) are flagged with a pound sign (#). Species that are elevated above the normal range in JBTS10 sample(s) are flagged with a caret (^). Species below the normal range in JBTS10 sample(s) are flagged with a dagger (†).
